# Supplementary material for: Whole genome sequence analysis of multi drug resistant community associated methicillin resistant Staphylococcus aureus from food fish: detection of clonal lineage ST 28 and its antimicrobial resistance and virulence genes
Source: PeerJ. 2021 May 24;9:e11224. doi: 10.7717/peerj.11224 (PMC8158172; doi:10.7717/peerj.11224)
Supplement: Supplemental Information 1 [file peerj-09-11224-s001.pdf]

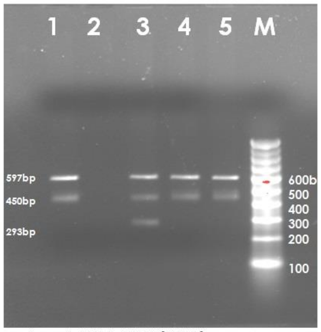


Lane 1: ATCC 25923 (MSSA)
Lane 2: Negative Control
Lane 3: ATCC 44330 (MRSA)
Lane 4: Sample No.1
Lane 5: Sample No.2
597bp: S.aureus genus specific
450bp: Fem A
293bp: Mec A
M: 100bp Ladder

**Supplementary Figure S1: Standardization of multiplex PCR Amplification of MRSA isolates with genus specific primers (16S rRNA, 597bp); femA (450bp) and mecA gene (293bp)**

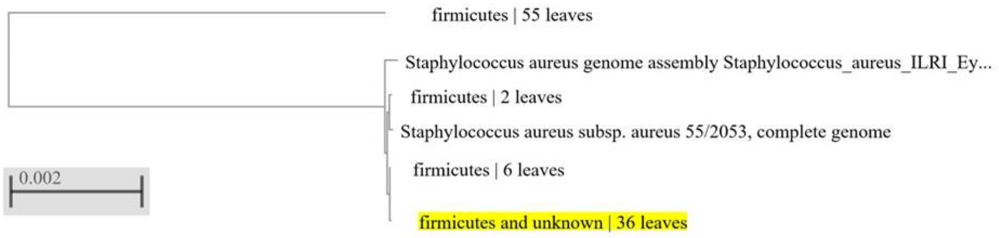


**Supplementary Figure S2: Phylogenetic tree constructed based on antimicrobial resistant genes (ARGs)**

**Supplementary Figure S3: Phylogenetic tree constructed based on virulence genes**

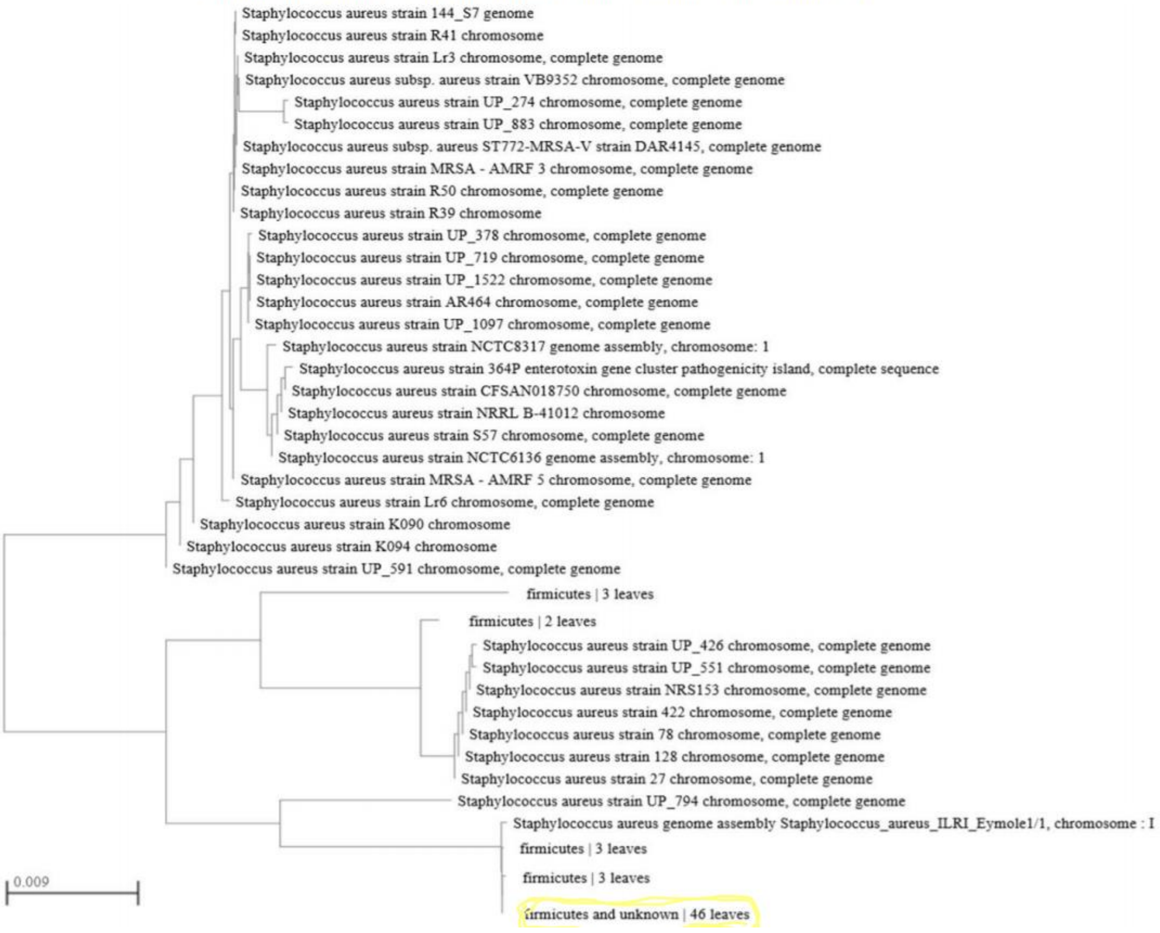


**Supplementary Figure S3: Phylogenetic tree constructed based on viulence genes**
