# Supplementary material for: Whole genome sequence analysis of multi drug resistant community associated methicillin resistant Staphylococcus aureus from food fish: detection of clonal lineage ST 28 and its antimicrobial resistance and virulence genes
Source: PeerJ. 2021 May 24;9:e11224. doi: 10.7717/peerj.11224 (PMC8158172; doi:10.7717/peerj.11224)
Supplement: Supplemental Information 2 [file peerj-09-11224-s002.pdf]

Table S1:

Whole Genome Sequence Project information

**Description**	**Value**
Finishing quality	Draft genome
Library used	2x 250 bp
Sequencing platforms	Illumina HiSeq 2500
Library type	Paired End
Project type	*De novo* genome assembly
Assembly method	MaSuRCA
Total number of paired-end Raw Reads	7,237,547
Genome coverage	544.0x
Total number of bases (Mb)	1447.5
GC content	35.11
Max Read Length	100
Min Read Length	100
High Quality data- No. of Reads	7,731,542
Total number of bases	731,267,948
Max Read Length	100
Min Read Length	95
GenBank ID	NBZX00000000.1
BioProject	PRJNA352109
BioSample	SAMN05967212
Source	Dried Ribbonfish

Table S2:

Nucleotide and gene contents levels of MRSA7 genome assembled *de novo* by MaSuRCA

**Description**	**Value**
Total Genome Length (bp)	2,851,163 (~2.8Mb)
No. of contigs	120
N50 length Scaffold (bp)	66,022
Average Scaffold length (bp)	23759.70
Largest Scaffold (bp)	170965
Smallest Scaffold (bp)	301
Number of predicted gene	2797
Number of predicted CDs with significant BLASTX match	2755 (98.49)
Number of predicted CDs with UniProt annotation	1741 (62.24)
Number of CDS with no significant BLASTX hit	40
Number of CDS with a significant BLASTX hit but no UniProt Information	1014
Average Gene Size	902
Longest Gene	30258
Shortest Gene	72
tRNA genes	80

Supplementary Table S3: Pathways Analysis of the Genes

Pathways	Number of genes	Number of Enzymes
Purine metabolism	168	44
Biosynthesis of antibiotics	14	132
Thiamine metabolism	118	9
Pyrimidine metabolism	33	23
Pyruvate metabolism	32	29
Glycine, serine and threonine metabolism	28	23
Glycolysis / Gluconeogenesis	28	23
Cysteine and methionine metabolism	26	22
Arginine biosynthesis	24	17
Amino sugar and nucleotide sugar metabolism	22	19
Aminoacyl-tRNA	21	17
Carbon fixation in photosynthetic organisms	16	13
Alanine, aspartate and glutamate metabolism	21	16
Propanoate metabolism	21	15
Pantothenate and CoA biosynthesis	18	15
Carbon fixation pathways in prokaryotes 17 16	17	16
Drug metabolism - other enzymes	17	7
Galactose metabolism	16	14
Aminobenzoate degradation	16	3
Phenylalanine, tyrosine and tryptophan biosynthesis	19	19
Methane metabolism	16	13
Carbon fixation in photosynthetic organisms	16	13
Pentose phosphate pathway	16	15

Citrate cycle (TCA cycle)	16	14
Arginine and proline metabolism	15	11
Terpenoid backbone biosynthesis	15	15
Butanoate metabolism	15	13
Lysine biosynthesis	14	11
Valine, leucine and isoleucine degradation	13	10
Histidine metabolism	13	12
Fructose and mannose metabolism	13	10
Nicotinate and nicotinamide metabolism	13	9
Nitrogen metabolism	13	9
Glyoxylate and dicarboxylate metabolism	12	13
Glutathione metabolism	12	7
Valine, leucine and isoleucine biosynthesis	12	8
Folate biosynthesis	12	12
Fatty acid biosynthesis	12	10

Enzyme Classification (EC) assignment was obtained using BLAST2GO functional class and orthologus assessment. Mapping of genes to the biological pathways were performed using BLAST2GO pro server. A total of 587 genes were mapped on different pathways out of which majority of the genes were falling in to the purine metabolism (168), Biosynthesis of antibiotics (145), and Thiamine metabolism (118)
